# *rac*-3-[(3-Chloro­anilino)(4-chloro­phenyl)meth­yl]thian-4-one

**DOI:** 10.1107/S1600536812004680

**Published:** 2012-02-10

**Authors:** Klaus Harms, M. Saeed Abaee, Mohammad M. Mojtahedi, A. Wahid Mesbah

**Affiliations:** a Fachbereich Chemie, Philipps Universität Marburg, Hans Meerwein Str., Marburg D-35032, Germany; b Department of Organic Chemistry, Chemistry and Chemical Engineering Research Center of Iran, PO Box 14335-186, Tehran, Iran

## Abstract

In the title compound, C_18_H_17_Cl_2_NOS, the thio­pyran­one ring adopts a chair conformation, with the substituent in the axial position. The dihedral angle between the two benzene rings is 89.43 (1)°. In the crystal, mol­ecules form inversion dimers through inter­molecular N—H⋯O hydrogen bonds [graph set *R*_2_^2^(8)].

## Related literature

For the preparation and spectroscopic characterization of a series of related compounds and the crystal structure of 3-[(phenyl­amino)(*p*-tol­yl)methly]dihydro-2*H*-thio­pyran-4(3*H*)-one, see: Abaee *et al.* (2012 ▶). For the crystal structures of related compounds, see: Guo *et al.* (2007 ▶); Fun *et al.* (2009 ▶); Harms *et al.* (2012 ▶). For patterns in hydrogen bonding, see: Bernstein *et al.* (1995 ▶). For defining the relative configuration of diastereomers, see: IUPAC (2012 ▶).
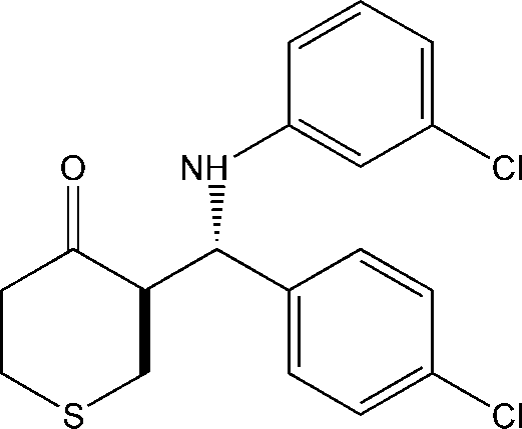


## Experimental

### 

#### Crystal data

C_18_H_17_Cl_2_NOS
*M* *_r_* = 366.29
Monoclinic, 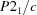

*a* = 11.2611 (8) Å
*b* = 8.6686 (7) Å
*c* = 18.0976 (12) Å
β = 105.266 (8)°
*V* = 1704.3 (2) Å^3^
*Z* = 4
Mo *K*α radiation
μ = 0.51 mm^−1^
*T* = 193 K
0.29 × 0.24 × 0.05 mm

#### Data collection

Stoe IPDS-1 image-plate diffractometer
Absorption correction: integration *X-RED32* (Stoe & Cie, 2006 ▶) *T*_min_ = 0.890, *T*_max_ = 0.978
15404 measured reflections
3134 independent reflections
1867 reflections with *I* > 2σ(*I*)
*R*_int_ = 0.042

#### Refinement

*R*[*F* ^2^ > 2σ(*F* ^2^)] = 0.024
*wR*(*F* ^2^) = 0.048
*S* = 0.64
3134 reflections
212 parameters
H atoms treated by a mixture of independent and constrained refinement
Δρ_max_ = 0.17 e Å^−3^
Δρ_min_ = −0.15 e Å^−3^

### 

Data collection: *EXPOSE* (Stoe & Cie, 1994 ▶); cell refinement: *CELL* (Stoe & Cie, 1994 ▶); data reduction: *X-RED32* (Stoe & Cie, 2006 ▶); program(s) used to solve structure: *SIR92* (Altomare *et al.*, 1994 ▶); program(s) used to refine structure: *SHELXL97* (Sheldrick, 2008 ▶); molecular graphics: *DIAMOND* (Brandenburg, 2007 ▶); software used to prepare material for publication: *publCIF* (Westrip, 2010 ▶), *PLATON* (Spek, 2009 ▶) and *WinGX* (Farrugia, 1999 ▶).

## Supplementary Material

Crystal structure: contains datablock(s) I, global. DOI: 10.1107/S1600536812004680/zs2176sup1.cif

Structure factors: contains datablock(s) I. DOI: 10.1107/S1600536812004680/zs2176Isup2.hkl

Supplementary material file. DOI: 10.1107/S1600536812004680/zs2176Isup3.cml

Additional supplementary materials:  crystallographic information; 3D view; checkCIF report

## Figures and Tables

**Table 1:** Hydrogen-bond geometry (Å, °)

*D*—H⋯*A*	*D*—H	H⋯*A*	*D*⋯*A*	*D*—H⋯*A*
N8—H8⋯O1^i^	0.81 (2)	2.27 (2)	3.070 (2)	168 (2)

Symmetry code: (i) 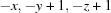.

**Fig. 1.**
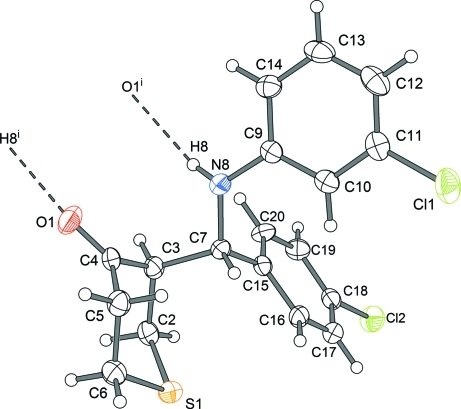
Molecular structure of the title compound with the atom numbering scheme with displacement ellipsoids drawn at 50% probability level. Dashed lines indicate hydrogen bonds to the neighbouring molecule generated by crystallographic inversion symmetry [for symmetry code (i), see Table 1].

## supplementary crystallographic information

### Comment

The title compound is an example of a product from an *anti*-selective three-component Mannich reaction involving the thiopyran-4-one system (Abaee *et al.*, 2012). The same *anti* configuration has been found recently in the crystal structures of two other compounds of this series (Abaee *et al.*, 2012; Harms *et al.*, 2012). In the title compund the thiopyranone ring adopts a chair-like conformation with the substituent in the axial position. The relative configuration of the stereogenic centres at C3 and C7 is R*, S* (IUPAC, 1997). The dihedral angle between the two benzene rings in the molecule is 89.43 (10)°. In the crystal packing the molecules form discrete centrosymmetric dimers are through intermolecular N—H···O hydrogen bonds [graph set *R*_2_^2^(8)] (Bernstein *et al.*, 1995). For further details see Table 1 and Fig. 1.

### Experimental

The title compound was synthesized using an *anti*-selective three-component Mannich reaction involving the thiopyran-4-one system (Abaee *et al.*, 2012). Colourless crystals suitable for the crystal structure determination were grown from ethyl acetate.

### Refinement

All C-bonded H atoms were placed in geometrical positions and constrained to ride on their parent atoms with C—H distances in the range 0.95–1.00 Å. The *U*_iso_ values were constrained to be 1.2*U*_eq_ of the parent C atom. The position of the N-bonded H atom has been refined freely with an isotropic displacement parameter. The N—H bond length is 0.81 (2) Å.

### Crystal data

**Table:**

C_18_H_17_Cl_2_NOS	*F*(000) = 760
*M**_r_* = 366.29	*D*_x_ = 1.428 Mg m^−^^3^
Monoclinic, *P*2_1_/*c*	Mo *K*α radiation, λ = 0.71073 Å
Hall symbol: -P 2ybc	Cell parameters from 8000 reflections
*a* = 11.2611 (8) Å	θ = 1.9–25°
*b* = 8.6686 (7) Å	µ = 0.51 mm^−^^1^
*c* = 18.0976 (12) Å	*T* = 193 K
β = 105.266 (8)°	Plate, colourless
*V* = 1704.3 (2) Å^3^	0.29 × 0.24 × 0.05 mm
*Z* = 4	

### Data collection

**Table:**

Stoe IPDS-1 image-plate diffractometer	3134 independent reflections
Radiation source: fine-focus sealed tube	1867 reflections with *I* > 2σ(*I*)
Graphite monochromator	*R*_int_ = 0.042
Detector resolution: 6.67 pixels mm^-1^	θ_max_ = 25.4°, θ_min_ = 1.9°
ω scans	*h* = −13→13
Absorption correction: integration *X-RED32* (Stoe & Cie, 2006)	*k* = −10→10
*T*_min_ = 0.890, *T*_max_ = 0.978	*l* = −21→21
15404 measured reflections	

### Refinement

**Table:**

Refinement on *F*^2^	Primary atom site location: structure-invariant direct methods
Least-squares matrix: full	Secondary atom site location: difference Fourier map
*R*[*F*^2^ > 2σ(*F*^2^)] = 0.024	Hydrogen site location: inferred from neighbouring sites
*wR*(*F*^2^) = 0.048	H atoms treated by a mixture of independent and constrained refinement
*S* = 0.64	*w* = 1/[σ^2^(*F*_o_^2^) + (0.0127*P*)^2^] where *P* = (*F*_o_^2^ + 2*F*_c_^2^)/3
3134 reflections	(Δ/σ)_max_ = 0.001
212 parameters	Δρ_max_ = 0.17 e Å^−^^3^
0 restraints	Δρ_min_ = −0.15 e Å^−^^3^

### Special details

**Table:**

Geometry. All e.s.d.'s (except the e.s.d. in the dihedral angle between two l.s. planes) are estimated using the full covariance matrix. The cell e.s.d.'s are taken into account individually in the estimation of e.s.d.'s in distances, angles and torsion angles; correlations between e.s.d.'s in cell parameters are only used when they are defined by crystal symmetry. An approximate (isotropic) treatment of cell e.s.d.'s is used for estimating e.s.d.'s involving l.s. planes.
Refinement. Refinement of *F*^2^ against ALL reflections. The weighted *R*-factor *wR* and goodness of fit *S* are based on *F*^2^, conventional *R*-factors *R* are based on *F*, with *F* set to zero for negative *F*^2^. The threshold expression of *F*^2^ > σ(*F*^2^) is used only for calculating *R*-factors(gt) *etc*. and is not relevant to the choice of reflections for refinement. *R*-factors based on *F*^2^ are statistically about twice as large as those based on *F*, and *R*-factors based on ALL data will be even larger.

### Fractional atomic coordinates and isotropic or equivalent isotropic displacement parameters (Å^2^)

**Table:**

	*x*	*y*	*z*	*U*_iso_*/*U*_eq_	
C2	0.31042 (19)	0.2357 (2)	0.53806 (11)	0.0272 (5)	
H2A	0.3860	0.2956	0.5399	0.033*	
H2B	0.2797	0.1939	0.4856	0.033*	
C3	0.21227 (18)	0.3451 (2)	0.55471 (10)	0.0238 (5)	
H3	0.1946	0.4265	0.5141	0.029*	
C4	0.09371 (19)	0.2559 (2)	0.54853 (11)	0.0260 (5)	
C5	0.10024 (19)	0.1153 (2)	0.59821 (11)	0.0282 (5)	
H5A	0.0184	0.0653	0.5864	0.034*	
H5B	0.1218	0.1472	0.6527	0.034*	
C6	0.19574 (19)	−0.0013 (2)	0.58604 (12)	0.0308 (5)	
H6A	0.1703	−0.0387	0.5325	0.037*	
H6B	0.1970	−0.0910	0.6201	0.037*	
C7	0.25464 (16)	0.4273 (2)	0.63362 (10)	0.0213 (4)	
H7	0.2599	0.3478	0.6744	0.026*	
C9	0.15786 (17)	0.6196 (2)	0.70276 (11)	0.0221 (4)	
C10	0.23976 (18)	0.5889 (2)	0.77388 (11)	0.0251 (4)	
H10	0.3020	0.5129	0.7783	0.030*	
C11	0.2297 (2)	0.6703 (2)	0.83810 (11)	0.0288 (5)	
C12	0.1428 (2)	0.7834 (3)	0.83454 (13)	0.0365 (6)	
H12	0.1373	0.8374	0.8792	0.044*	
C13	0.06325 (19)	0.8161 (3)	0.76353 (13)	0.0353 (5)	
H13	0.0032	0.8948	0.7594	0.042*	
C14	0.07008 (18)	0.7357 (2)	0.69860 (12)	0.0277 (5)	
H14	0.0145	0.7598	0.6507	0.033*	
C15	0.38184 (17)	0.4989 (2)	0.64544 (10)	0.0215 (4)	
C16	0.48456 (17)	0.4248 (2)	0.69185 (10)	0.0240 (4)	
H16	0.4738	0.3333	0.7182	0.029*	
C17	0.60194 (19)	0.4826 (2)	0.70002 (11)	0.0266 (5)	
H17	0.6714	0.4305	0.7312	0.032*	
C18	0.61680 (18)	0.6159 (2)	0.66259 (10)	0.0242 (4)	
C19	0.51657 (18)	0.6941 (2)	0.61697 (11)	0.0278 (5)	
H19	0.5278	0.7872	0.5919	0.033*	
C20	0.39949 (18)	0.6339 (2)	0.60864 (11)	0.0266 (5)	
H20	0.3303	0.6861	0.5772	0.032*	
N8	0.15854 (16)	0.5356 (2)	0.63804 (10)	0.0260 (4)	
O1	−0.00210 (14)	0.29351 (18)	0.50276 (9)	0.0404 (4)	
S1	0.34870 (5)	0.07723 (6)	0.60476 (3)	0.02864 (13)	
Cl1	0.33192 (6)	0.62648 (7)	0.92643 (3)	0.04561 (17)	
Cl2	0.76515 (5)	0.68900 (7)	0.67408 (3)	0.03580 (14)	
H8	0.1180 (19)	0.569 (2)	0.5974 (11)	0.027 (6)*	

### Atomic displacement parameters (Å^2^)

**Table:**

	*U*^11^	*U*^22^	*U*^33^	*U*^12^	*U*^13^	*U*^23^
C2	0.0317 (12)	0.0281 (12)	0.0222 (10)	−0.0019 (10)	0.0076 (9)	−0.0037 (9)
C3	0.0298 (11)	0.0228 (11)	0.0172 (10)	0.0003 (9)	0.0031 (9)	0.0016 (8)
C4	0.0279 (12)	0.0285 (12)	0.0185 (10)	0.0027 (9)	0.0009 (9)	−0.0070 (9)
C5	0.0262 (11)	0.0277 (12)	0.0297 (11)	−0.0043 (10)	0.0058 (9)	0.0001 (10)
C6	0.0331 (12)	0.0244 (11)	0.0335 (12)	−0.0026 (10)	0.0061 (10)	−0.0013 (10)
C7	0.0233 (10)	0.0211 (10)	0.0184 (9)	0.0029 (9)	0.0035 (8)	0.0015 (9)
C9	0.0196 (10)	0.0201 (11)	0.0283 (10)	−0.0057 (9)	0.0095 (9)	−0.0017 (9)
C10	0.0255 (11)	0.0216 (11)	0.0307 (11)	−0.0034 (9)	0.0116 (9)	−0.0013 (9)
C11	0.0309 (12)	0.0298 (13)	0.0284 (11)	−0.0103 (10)	0.0125 (10)	−0.0022 (10)
C12	0.0368 (13)	0.0357 (14)	0.0444 (14)	−0.0102 (11)	0.0239 (11)	−0.0131 (11)
C13	0.0263 (12)	0.0308 (13)	0.0533 (14)	0.0015 (11)	0.0186 (11)	−0.0070 (11)
C14	0.0209 (11)	0.0262 (12)	0.0369 (12)	−0.0015 (9)	0.0095 (9)	0.0000 (10)
C15	0.0255 (11)	0.0220 (10)	0.0166 (10)	0.0021 (9)	0.0045 (8)	−0.0024 (9)
C16	0.0281 (11)	0.0220 (11)	0.0199 (9)	0.0000 (10)	0.0026 (8)	0.0017 (9)
C17	0.0262 (11)	0.0270 (12)	0.0243 (10)	0.0033 (10)	0.0026 (9)	−0.0021 (9)
C18	0.0241 (11)	0.0244 (11)	0.0245 (10)	−0.0013 (9)	0.0072 (8)	−0.0060 (9)
C19	0.0314 (12)	0.0210 (11)	0.0319 (11)	0.0001 (10)	0.0101 (10)	0.0048 (10)
C20	0.0240 (11)	0.0234 (11)	0.0312 (11)	0.0061 (9)	0.0053 (9)	0.0052 (10)
N8	0.0254 (10)	0.0282 (11)	0.0222 (9)	0.0053 (8)	0.0025 (8)	−0.0009 (8)
O1	0.0317 (8)	0.0408 (9)	0.0384 (8)	0.0017 (8)	−0.0090 (7)	0.0025 (8)
S1	0.0268 (3)	0.0258 (3)	0.0316 (3)	0.0030 (3)	0.0046 (2)	0.0003 (2)
Cl1	0.0576 (4)	0.0523 (4)	0.0252 (3)	−0.0036 (3)	0.0077 (3)	−0.0042 (3)
Cl2	0.0269 (3)	0.0373 (3)	0.0424 (3)	−0.0062 (3)	0.0076 (2)	−0.0012 (3)

### Geometric parameters (Å, º)

**Table:**

C2—C3	1.545 (3)	C10—C11	1.389 (3)
C2—S1	1.804 (2)	C10—H10	0.9500
C2—H2A	0.9900	C11—C12	1.375 (3)
C2—H2B	0.9900	C11—Cl1	1.748 (2)
C3—C4	1.521 (3)	C12—C13	1.388 (3)
C3—C7	1.555 (2)	C12—H12	0.9500
C3—H3	1.0000	C13—C14	1.385 (3)
C4—O1	1.219 (2)	C13—H13	0.9500
C4—C5	1.505 (3)	C14—H14	0.9500
C5—C6	1.533 (3)	C15—C20	1.386 (3)
C5—H5A	0.9900	C15—C16	1.395 (3)
C5—H5B	0.9900	C16—C17	1.385 (3)
C6—S1	1.799 (2)	C16—H16	0.9500
C6—H6A	0.9900	C17—C18	1.372 (3)
C6—H6B	0.9900	C17—H17	0.9500
C7—N8	1.451 (2)	C18—C19	1.388 (3)
C7—C15	1.524 (3)	C18—Cl2	1.747 (2)
C7—H7	1.0000	C19—C20	1.389 (3)
C9—N8	1.381 (2)	C19—H19	0.9500
C9—C10	1.397 (3)	C20—H20	0.9500
C9—C14	1.399 (3)	N8—H8	0.81 (2)
			
C3—C2—S1	113.20 (13)	C11—C10—H10	120.2
C3—C2—H2A	108.9	C9—C10—H10	120.2
S1—C2—H2A	108.9	C12—C11—C10	122.5 (2)
C3—C2—H2B	108.9	C12—C11—Cl1	119.15 (16)
S1—C2—H2B	108.9	C10—C11—Cl1	118.39 (17)
H2A—C2—H2B	107.8	C11—C12—C13	117.82 (19)
C4—C3—C2	109.45 (16)	C11—C12—H12	121.1
C4—C3—C7	110.60 (15)	C13—C12—H12	121.1
C2—C3—C7	113.47 (15)	C14—C13—C12	121.1 (2)
C4—C3—H3	107.7	C14—C13—H13	119.5
C2—C3—H3	107.7	C12—C13—H13	119.5
C7—C3—H3	107.7	C13—C14—C9	120.82 (19)
O1—C4—C5	121.19 (19)	C13—C14—H14	119.6
O1—C4—C3	121.12 (19)	C9—C14—H14	119.6
C5—C4—C3	117.63 (17)	C20—C15—C16	118.50 (18)
C4—C5—C6	111.94 (16)	C20—C15—C7	121.65 (17)
C4—C5—H5A	109.2	C16—C15—C7	119.80 (17)
C6—C5—H5A	109.2	C17—C16—C15	120.98 (19)
C4—C5—H5B	109.2	C17—C16—H16	119.5
C6—C5—H5B	109.2	C15—C16—H16	119.5
H5A—C5—H5B	107.9	C18—C17—C16	119.29 (19)
C5—C6—S1	113.37 (15)	C18—C17—H17	120.4
C5—C6—H6A	108.9	C16—C17—H17	120.4
S1—C6—H6A	108.9	C17—C18—C19	121.32 (19)
C5—C6—H6B	108.9	C17—C18—Cl2	118.95 (16)
S1—C6—H6B	108.9	C19—C18—Cl2	119.73 (16)
H6A—C6—H6B	107.7	C18—C19—C20	118.74 (19)
N8—C7—C15	114.59 (17)	C18—C19—H19	120.6
N8—C7—C3	107.13 (14)	C20—C19—H19	120.6
C15—C7—C3	111.44 (14)	C15—C20—C19	121.15 (19)
N8—C7—H7	107.8	C15—C20—H20	119.4
C15—C7—H7	107.8	C19—C20—H20	119.4
C3—C7—H7	107.8	C9—N8—C7	123.97 (17)
N8—C9—C10	122.09 (18)	C9—N8—H8	116.9 (15)
N8—C9—C14	119.70 (18)	C7—N8—H8	115.6 (14)
C10—C9—C14	118.17 (18)	C6—S1—C2	96.53 (10)
C11—C10—C9	119.63 (19)		
			
S1—C2—C3—C4	−61.14 (18)	C10—C9—C14—C13	−1.2 (3)
S1—C2—C3—C7	62.93 (19)	N8—C7—C15—C20	−46.3 (2)
C2—C3—C4—O1	−120.6 (2)	C3—C7—C15—C20	75.5 (2)
C7—C3—C4—O1	113.7 (2)	N8—C7—C15—C16	136.32 (18)
C2—C3—C4—C5	56.6 (2)	C3—C7—C15—C16	−101.83 (19)
C7—C3—C4—C5	−69.1 (2)	C20—C15—C16—C17	−1.2 (3)
O1—C4—C5—C6	121.5 (2)	C7—C15—C16—C17	176.23 (17)
C3—C4—C5—C6	−55.7 (2)	C15—C16—C17—C18	0.8 (3)
C4—C5—C6—S1	58.2 (2)	C16—C17—C18—C19	0.3 (3)
C4—C3—C7—N8	−61.7 (2)	C16—C17—C18—Cl2	179.32 (15)
C2—C3—C7—N8	174.84 (16)	C17—C18—C19—C20	−0.9 (3)
C4—C3—C7—C15	172.20 (16)	Cl2—C18—C19—C20	180.00 (15)
C2—C3—C7—C15	48.8 (2)	C16—C15—C20—C19	0.5 (3)
N8—C9—C10—C11	−175.75 (18)	C7—C15—C20—C19	−176.88 (18)
C14—C9—C10—C11	1.9 (3)	C18—C19—C20—C15	0.5 (3)
C9—C10—C11—C12	−1.3 (3)	C10—C9—N8—C7	−10.8 (3)
C9—C10—C11—Cl1	178.88 (15)	C14—C9—N8—C7	171.62 (18)
C10—C11—C12—C13	−0.2 (3)	C15—C7—N8—C9	−62.2 (2)
Cl1—C11—C12—C13	179.69 (16)	C3—C7—N8—C9	173.59 (17)
C11—C12—C13—C14	0.9 (3)	C5—C6—S1—C2	−56.47 (16)
C12—C13—C14—C9	−0.3 (3)	C3—C2—S1—C6	58.68 (16)
N8—C9—C14—C13	176.54 (18)		

### Hydrogen-bond geometry (Å, º)

**Table:**

*D*—H···*A*	*D*—H	H···*A*	*D*···*A*	*D*—H···*A*
N8—H8···O1^i^	0.81 (2)	2.27 (2)	3.070 (2)	168 (2)

Symmetry code: (i) −*x*, −*y*+1, −*z*+1.
